# Research highlights, *The Plant Genome*, Volume 16, Issue 2

**DOI:** 10.1002/tpg2.20359

**Published:** 2023-05-31

## 1 CLONING TOOLKIT STREAMLINES VECTOR DESIGN

DNA vectors encoding gene expression cassettes are a key tool in plant biotechnology. Complex vectors with multiple cassettes can be challenging to assemble through molecular cloning and often have divergent requirements for expression once delivered into host‐plant cells. Chamness et al. (https://doi.org/10.1002/tpg2.20312) presented a vector toolkit that streamlines the assembly of complex vectors using efficient cloning reactions and a large library of genetic parts. The library includes both protein‐coding genes and regulatory elements controlling gene expression. Part selection for new cassettes is aided by benchmarking experiments that quantify expression from different regulatory elements.

## 2 REDUCED UREIDE BIOSYNTHESIS INDUCES NODULE SENESCENCE

Soybean nodulation is the result of a symbiotic interaction between soybean roots and soil bacteria (rhizobia), which provides the soybean plant with a source of nitrogen in the form of the ureides allantoin and allantoate. Although the presence of the ureide biosynthesis pathway is well documented in legume plants, identification of the enzymes involved is largely based on predicted genome annotations with little direct genetic or biochemical evidence to confirm such predictions. Nguyen et al. (https://doi.org/10.1002/tpg2.20172) demonstrated that GmXDH1 (XANTHINE DEHYDROGENASE 1) and GmUOX1 (URICASE1) are the major isoforms catalyzing the formation of uric acid and allantoin, respectively, during ureide biosynthesis in soybean nodules. Moreover, the authors found that loss‐of‐function mutations in GmXDH1 and GmUOX1 resulted in early nodule senescence and induction of plant defense response. These results support the notion that early nodule senescence is not simply due to nitrogen deficiency caused by ineffective nitrogen fixation per se, but rather a reflection of active induction of plant defense response to non‐fixing symbiosis that confer no benefit to the host plant.

## 3 GRASS COPES WITH GENOME LOSS

Switchgrass is being bred for bioenergy production, and high biomass is one important consideration. If homozygous switchgrass lines were readily available, development of their hybrids with higher biomass would be feasible. Homozygous lines would also facilitate selection for other desirable traits as well as biotech crop improvement strategies. Yoon et al. (https://doi.org/10.1002/tpg2.20209) have made significant progress in this direction by adopting a strategy demonstrated in model plants that results in the elimination of one parent's genome to create individuals with half the normal set of chromosomes. These can then be chemically treated to form complete homozygotes. The process relies on the modification of a DNA‐binding histone variant called CENH3 that normally binds the chromosome's centromeres and helps bring them together with the mitotic spindle apparatus to ensure faithful chromosome replication. By crossing wild‐type switchgrass to transgenic lines carrying CENH3 gene modifications, the group was able to isolate individuals that had whole or partial genome elimination at significant frequencies. Those individuals with complete genome elimination were reduced in stature relative to wild‐type. However, they may form the future basis for efficient, completely homozygous, and switchgrass production.

## 4 PLANT VIRUS ROLE IN CRISPR‐CAS

Plant genome editing using CRISPR‐Cas technologies holds an unprecedented excitement for food security. However, efficient tools to deliver CRISPR‐Cas reaction components into plants are needed. Uranga and Daròs (https://doi.org/10.1002/tpg2.20220) reviewed the dual role of plant viruses in CRISPR‐Cas genome editing. On the one side, plant viruses are being repurposed into efficient tools to facilitate genome editing. On the other side, viral genomes or the host genes that virus require for infection are the targets to create new cultivars resistant to disease.

## 5 GENE EDITING FOR HEALTHIER RICE

Resistant starch may be beneficial to improve human health and reduce the risk of diet‐related chronic diseases. However, rice has low levels of resistant starch. Biswas et al. (https://doi.org/10.1002/tpg2.20225) reported rice with 15% higher levels of resistant starch using CRISPR‐Cas9 gene editing technology. Multiplex CRISPR‐Cas9 editing was performed to knock‐out the four starch branching enzymes in rice in the U.S. rice cultivar Presidio. This study demonstrates the potential of multiplex genome editing to develop rice lines with higher levels of resistant starch.

## 6 OFF‐TARGET EFFECTS OF MULTIPLEXED CRISPR‐CAS12A EDITING

Despite widespread use of CRISPR‐Cas systems for genome editing in crops, off‐target effects of multiplexed editing remain unclear. Using whole genome sequencing, Zhang et al. (https://doi.org/10.1002/tpg2.20266) investigated the off‐target effects of CRISPR‐Cas12a mediated multiplexed editing in rice. Large chromosomal rearrangement events such as deletion and translocation were discovered in some edited rice plants that endured more than 50 simultaneous DNA double strand breaks per diploid cell, but not in edited plants that had fewer than 10 DNA double strand breaks. The results suggest that the likelihood of chromosomal rearrangements increases when the number of DNA double strand breaks increases in multiplexed editing. This study shed light on the analysis and regulation of engineered crops derived from CRISPR‐Cas mediated multiplexed genome editing.

## 7 IMPROVING ALFALFA THROUGH GENOMICS AND ENVIROMICS

More alfalfa (*Medicago sativa* L.) is produced in the United State than in any other country, but alfalfa yield has been stagnant for decades. We need to breed new varieties for yield and novel techniques will help us speed up this process. Genomics and enviromics can be implemented in plant breeding pipelines to shorten the time between crossing and variety releases. DNA markers spread across the alfalfa genome can track beneficial gene forms and warrant their presence in successive generations. However, genomic data alone may not provide enough information to predict complex traits due to the presence of genotype by environment interactions. Enviromics can enhance prediction models by integrating environmental data into genomic‐based models. In Florida, nondormant alfalfa can grow year‐round but abiotic and biotic stresses during summer result in yield reduction and plant mortality. Cooler temperature allows for optimal growth during fall and spring, but drought conditions are common at these times. Fernandes et al. (https://doi.org/10.1002/tpg2.20306) studied the seasonal yield distribution of alfalfa in Florida, and enviromics enhanced genomic prediction models by modeling genotype by environment interactions. The study demonstrated the importance of including multilayered omics data to increase the predictive ability of complex traits in alfalfa.

## 8 DOUBLED HAPLOIDS HAVE FREQUENT CHROMOSOME ABERRATIONS

Doubled haploids (DH) are important for accelerating plant breeding efforts, by generating completely uniform inbred lines in a single generation. Contrary this prevalent understanding, Shrestha et al. (https://doi.org/10.1002/tpg2.20309) observed high heterogeneity within DH lines from two wheat populations. They observed a large range of chromosome aberrations, which included chromosome deletions, duplications, and various aneuploidies. Strikingly, the frequency of aberrant DH lines was high, ranging from 45% to 55% of the population. With the prevalent use of DH in plant breeding, chromosome aberrations and heterogeneity have important implications for how to use these materials for crop improvement.

## 9 HOW MAIZE RESISTS HUNGRY CATERPILLARS

Maize (corn) is a crop of major economic and food security importance globally, and the fall armyworm (FAW) can devastate entire maize crops, especially in countries or markets that do not allow the use of transgenic crops. Host‐plant insect resistance conferred by naturally occurring maize genes is an economical and environmentally benign way to control FAW. Warburton et al. (https://doi.org/10.1002/tpg2.20311) grew 289 different kinds of maize in the field and put FAW larvae on them and then measured damage to the leaves. They identified 30 maize lines that showed significantly less damage than the rest. We also identified seven genes and several metabolic pathways, the mechanisms that cells use to build important proteins and chemicals, that were associated with reduced insect feeding damage. We can now use this information to breed new, resistant maize varieties for farmers.

## 10 ONT‐BASED IN‐DEL DETECTION IN CROPS

Structural variations are larger polymorphisms that can have a strong impact on crop traits. Comprehensive SVs detection in many crop genomes remains challenging, hindered by their size and complexity. Yildiz et al. (https://doi.org/10.1002/tpg2.20314) used real‐world and simulated data representing diploid and polyploidy crops to benchmark performance of SV detection tools. They test 20 long read aligner and SV discovery tool combinations for the identification of insertions and deletions with Oxford Nanopore Technology (ONT) at low to medium coverages (5× to 20×). These benchmarks provide a useful guide for designing ONT re‐sequencing projects and implementing SV discovery pipelines in crop plant studies.

## 11 IGTCM DATABASE PROMOTES TCM VALUE MINING

Active ingredient in Medicinal plants is various and became a vast natural product library. Gene annotation, storage, visualization, and comparative genomics analysis of medicinal plant genomes are the basis for exploring their medicinal value. In a new study by Ye et al. (https://doi.org/10.1002/tpg2.20317), all published genomic data (whole genome sequence, gene annotation, coding sequence, RNA sequence, amino acid sequence, and active ingredient) of medicinal plants were collected from public databases, and corresponding proteins and pathways were annotated with eggNOG‐mapper tool. On this basis, they built a comprehensive database used for storage and analysis of 83 medicinal plant genomes on the website IGTCM (http://yeyn.group:96) which is embedded with commonly used genome analysis tools such as BLAST and JBrowse. The establishment of IGTCM not only provides an analytical platform for the study of genomic structure and function of medicinal plants but also has important practical significance and value for the development of medicinal plant resources.

## 12 HOMOZYGOSITY MAPPING IDENTIFIES FREEZING‐TOLERANCE GENES

Homozygosity mapping is a new sequence‐based method to identify genomic region responsible for a given trait when the phenotype is controlled by a small number of dominant or codominant loci in plants. Whole genome homozygosity mapping by Shaikh et al. (https://doi.org/10.1002/tpg2.20318) identified 9 genomic regions and 22 candidate genes on a portion of chromosome 11 that were associated with freezing tolerance differences between two divergent *Camelina sativa* genotypes with very different freezing tolerances. This data combined with differential expression analysis highlighted a *cysteine‐rich RLKs*, *receptor serine/threonine kinases* and *rotamase cyclophilin 2* as the best candidates for freezing tolerance differences between these two genotypes.

## 13 GENE CO‐EXPRESSION ANALYSIS USING TIDYVERSE AND GRAPH‐BASED CLUSTERING

Gene co‐expression analysis has become an integral tool for plant genomics. Numerous publications have utilized gene co‐expression analyses, in which a wide range of plant species were studied, including Arabidopsis and many important crop species. Li et al. (https://doi.org/10.1002/tpg2.20323) described a gene co‐expression workflow in the R programing language. The workflow is built upon (1) the tidyverse, a collection of packages that provide user‐friendly solutions for data analysis and visualization, and (2) igraph, a package designed for networked analysis. The workflow, which has been benchmarked with two distinct plant transcriptome datasets, is highly customizable across multiple stages of the pipeline and generates publication quality figures.

## 14 GENOMIC SELECTION IMPROVES POTATO YIELD

The breeding progress for potato yield is low due to its tetraploidy, its heterozygosity as well as its low multiplication coefficient. Potato breeding programs could highly benefit from genomic selection (GS), which would allow selection at early stages on yield when its phenotypic evaluation is not possible. Wu et al. (https://doi.org/10.1002/tpg2.20327) demonstrated that integrating GS in a typical potato breeding can improve the short‐term gain compared to phenotypic selection. In addition, they show how implementing GS in consecutive selection stages can enhance short‐term genetic gain and recommend the breeders to perform GS at single hills and A clone stages. Furthermore, the optimal implementation of GS requires changes in the allocations of resources by adjusting the selection intensities compared to those typically used in phenotypically driven breeding programs. Therefore, this study provides new insight for potato breeders regarding how to optimally implement GS and improve the short‐term genetic gain for their target trait.

## 15 HAIRY VETCH RNA SEQUENCING, SEED DORMANCY RELATED GENES

Hairy vetch (*Vicia villosa* Roth) is a cool season legume with broad adaptability, winter hardiness, and vigorous growth habit. It has a potential to use as forage and as cover crop. Despite its impressive performance, seed dormancy/hardness and pod shattering are major constraints for growing hairy vetch commercially. mRNA sequencing of different hairy vetch tissues was performed by Ali et al. (https://doi.org/10.1002/tpg2.20330) to identify candidate genes associated with seed dormancy/hardness, pod shattering, and other important agronomic traits with their relative expression levels. Identification of these interesting candidate genes will facilitate the development of improved cultivars with desirable seed characteristics. These genes might also be target for manipulations through advanced technology such as genome editing for the development of novel germplasm for hairy vetch.

## 16 EXPLORING SOYBEAN OIL GENE DIVERSITY

Soybean is the most widely produced oil crop globally. Breeding new soybean varieties that produce oil with better fatty acid profiles is challenged by a lack of genetic diversity in fatty acid biosynthesis genes. Using the thousands of soybean genomes that have recently been published, Derbyshire et al. (https://doi.org/10.1002/tpg2.20334) comprehensively documented genetic diversity in soybean fatty acid biosynthesis genes. New genomic variation in these genes involves polymorphisms with moderate to high impacts on enzyme function. This research is an important reference for investigations into fatty acid biosynthesis variability between soybean varieties.

## 17 GENOMIC REGIONS FOR GRAIN QUALITY

Grain quality traits are the key factors that determine the economic value of wheat and highly governed by a type of quantitative genetic basis. Li et al. (https://doi.org/10.1002/tpg2.20336) reported that key genomic regions and putative candidate genes for wheat grain quality traits were identified by meta‐QTL analysis and in silico transcriptome integration. The 313 initial QTLs were refined into 64 meta‐QTLs. The average confidence interval of the meta‐QTLs was 2.49‐fold less than that of the initial QTLs. In combination with transcriptional and omics analyses, 135 putative candidate genes were identified from 64 MQTL regions.

## 18 GENETIC LOCI REGULATING THE CONCENTRATIONS OF ANTHOCYANINS AND PROANTHOCYANIDINS IN THE PERICARPS OF PURPLE AND RED RICE

Making rice colorfully healthy, anthocyanins and proanthocyanidins are the pigmented antioxidant flavonoids that give blueberries, cranberries, and red wine their color and health‐beneficial attributes. The syntheses of anthocyanins and proanthocyanidins in the outer layers of purple and red rice grains are known to be turned on by the Pb and Rc genes, respectively. Chen et al. (https://doi.org/10.1002/tpg2.20338) identified chromosome regions containing genes that further increase the concentrations of these health‐beneficial pigmented compounds and determined that combining the Pb and Rc genes mutually increased both anthocyanins and proanthocyanidins. This study advances knowledge on gene regulation of pigmented flavonoids and provides molecular markers useful for marker‐assisted breeding to develop rice cultivars with enhanced nutritional quality.

## 19 GENETIC SELECTION WHILE INBREEDING POTATO

Because of its autotetraploid genetic structure, commercial potato can only be maintained and distributed asexually, either in tissue culture or as tubers, which is inefficient for breeding and seed production. To overcome these limitations, potato breeders worldwide are working to develop diploid, inbred lines, and ultimately F_1_ hybrid varieties. Song and Endelman (https://doi.org/10.1002/tpg2.20339) used genome‐wide markers and recent advances in statistical genetics to track the inheritance of founder chromosome segments (i.e., haplotypes) during inbreeding. In the first breeding cycle, genotypic selection for homozygosity at the self‐compatibility gene (*Sli*) was combined with phenotypic selection for plant vigor, fertility, and yield. In the next cycle, genotypic and phenotypic selections were used to identify a vigorous F_2_ individual homozygous for self‐compatibility and early maturity (*StCDF1.3*). Future selection cycles will focus on reaching higher levels of overall homozygosity and expanding the set of gene targets (e.g., for tuber shape, skin color, and disease resistance).

## 20 JUJUBE SUGAR CHANGE MECHANISMS REVEALED

Rainproof cultivation is commonly used to avoid rainfall damage during fruit harvest, but its effects on jujube sugar content and underlying molecular mechanisms are unknown. Ji et al. (https://doi.org/10.1002/tpg2.20341) found that the sugar content of jujube fruits was significantly higher under rainproof cultivation. Transcriptomic analysis revealed that rainproof cultivation enhanced the intrinsic metabolic activity of fruit development and identified four genes that regulate sugar content changes. The authors suggest that temperature, humidity, and moisture conditions were key factors affecting sugar accumulation. Overall, this study provides insights into the mechanisms underlying sugar regulation in jujube fruits grown under rainproof cultivation.

## 21 EFFICACY OF PLANT BREEDING USING GENOMIC INFORMATION

GS has been widely accepted and applied to plant and animal breeding. Montesinos‐López et al. (https://doi.org/10.1002/tpg2.20346) studied several datasets to respond to the practical question of whether the accuracy of genomic prediction increases when considering genomic as compared with not using genomic. We found across traits, environments, datasets, and metrics, that the average gain in prediction accuracy when genomic information is considered was 26.31%, whereas only in terms of Pearson's correlation, the gain was of 46.1%. If the quality of the makers and relatedness of the individuals increase, major gains in prediction accuracy can be obtained, but if these two factors decrease, a lower increase was achieved. Our findings reinforce genomic is vital for improving the prediction accuracy and therefore the realized genetic gain in genomic‐assisted plant breeding programs.

